# Asynchronous Magnetic Bead Rotation (AMBR) Microviscometer for Label-Free DNA Analysis

**DOI:** 10.3390/bios4010076

**Published:** 2014-03-21

**Authors:** Yunzi Li, David T. Burke, Raoul Kopelman, Mark A. Burns

**Affiliations:** 1 Department of Chemical Engineering, University of Michigan, Ann Arbor, MI 48109, USA; E-Mail: yunzili@umich.edu; 2 Department of Human Genetics, University of Michigan, Ann Arbor, MI 48109, USA; E-Mail: dtburke@umich.edu; 3 Department of Chemistry, University of Michigan, Ann Arbor, MI 48109, USA; 4 Department of Biomedical Engineering, University of Michigan, Ann Arbor, MI 48109, USA

**Keywords:** label-free, DNA, quantitative PCR, restriction digestion, genetic analysis, viscosity, viscometer, magnetic, paramagnetic, microdevice

## Abstract

We have developed a label-free viscosity-based DNA detection system, using paramagnetic beads as an asynchronous magnetic bead rotation (AMBR) microviscometer. We have demonstrated experimentally that the bead rotation period is linearly proportional to the viscosity of a DNA solution surrounding the paramagnetic bead, as expected theoretically. Simple optical measurement of asynchronous microbead motion determines solution viscosity precisely in microscale volumes, thus allowing an estimate of DNA concentration or average fragment length. The response of the AMBR microviscometer yields reproducible measurement of DNA solutions, enzymatic digestion reactions, and PCR systems at template concentrations across a 5000-fold range. The results demonstrate the feasibility of viscosity-based DNA detection using AMBR in microscale aqueous volumes.

## 1. Introduction

Sensitive and cost-effective DNA detection methods have a wide range of applications, from clinical diagnostics and drug development to the food industry and forensic sciences [1,2,3,4]. In medical diagnostics, especially for infectious diseases, DNA detection technology such as quantitative polymerase chain reaction (qPCR), restriction fragment length polymorphism (RFLP) and ligation detection reaction (LDR) are crucial diagnostic tools [5,6,7]. Fluorescence has been used almost exclusively as the DNA detection method in these tools due to its simplicity and high sensitivity [6,7]. Recently, numerous efforts have been made to seek more cost-effective DNA detection technologies, notably for use in the developing world, yet none of them achieve the same sensitivity and applicability as fluorescence-based methods [8,9,10].

Another approach to detect and quantify DNA in diagnostic reactions is to measure the solution viscosity [11,12]. The viscosity of a double-stranded DNA (dsDNA) solution at a known temperature depends on the mass concentration and the average length of the DNA strands. The solution viscosity can indicate DNA concentration and/or length [13,14,15]. In restriction digestion reactions the solution viscosity decreases as longer DNA strands are cut into shorter pieces. Alternatively, in PCR, the solution viscosity increases as the length of the DNA increases, through polymerization of the target sequence. Notably, measurement of changes in solution viscosity does not require specific DNA chemical modification or pre-labeling.

Here we report on a microviscometer that is based on asynchronous magnetic bead rotation (AMBR) and used for DNA detection. AMBR detection monitors the rotational motion of a free-floating magnetic bead placed in a rotating magnetic field and uses changes in this motion to infer physical properties of the surrounding solution. When the rotation rate of the external field exceeds a critical value, the bead rotates at a speed different from that of the external field. The rate of this asynchronous rotation is viscosity dependent. Readily available paramagnetic microbeads can thus be used for measuring changes in DNA concentrations or average lengths.

## 2. Experimental Section

### 2.1. Reagents

Solutions used in the viscosity test were purchased from Sigma-Aldrich, unless otherwise specified. Samples tested in the experiment include glycerol and water solutions, lambda DNA *Eco*RI digest with lengths of 3530–21,226 bp, and pUC18 *Hae*III digest with lengths of 80–587 bp. Magnetic beads with diameters of 7.6, 16 and 45 μm were purchased from Spherotech Inc.

In digestion reactions and PCR amplification, lambda DNA was used as the template and purchased from Life Technologies. The restriction enzymes *Eco*RI with *Eco*RI buffer and *Pvu*I with NEBuffer 3 were purchased from New England Biolabs. For PCR, the forward primer is 5'-GGTGCTTTATGACTCTGCCGC-3', and the reverse primer is 5'-CGGCACTGGCAAGCAACTGA-3'. Both primers were purchased from Integrated DNA Technologies. PCR master mix was purchased from Promega.

### 2.2. Viscosity Measurement

The magnetic beads were washed with water three times and a concentrated bead solution was added to the samples (with 0.2% bovine serum albumin as a non-specific blocking agent). The bead concentration in the sample solution is 0.0075%w/v. The sample solution was rapidly mixed and then placed between two glass slides. The microviscometer can work with very small liquid volumes (<10 µL). Double-sided tape was inserted between the two glass slides, and nickel particles (210–420 μm) were placed on the edges of the tape to ensure a minimum gap of 210 μm between the two glass slides. Finally, silicone sealant (Dow Corning) was applied to the exterior edges to prevent sample evaporation.

**Figure 1:**
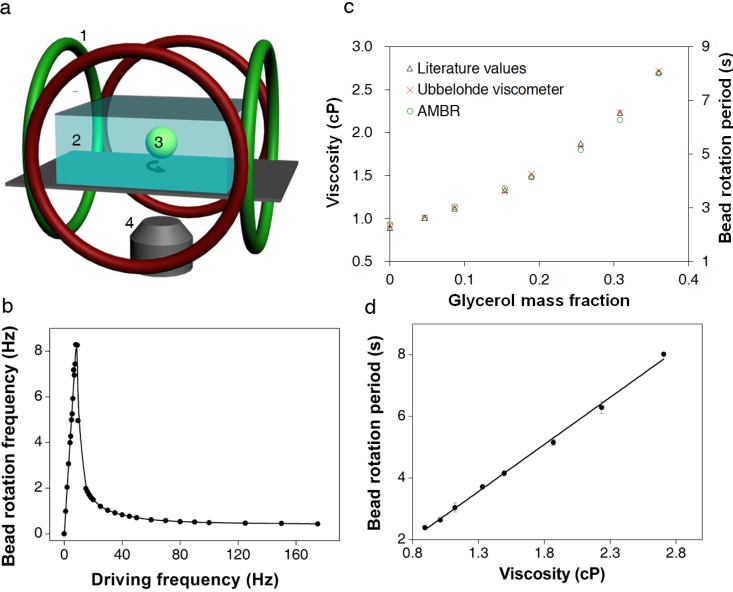
Asynchronous magnetic bead rotation (AMBR) microviscometer. (**a**) A schematic experimental set-up of an AMBR microviscometer. 1: perpendicular Helmholtz coils for rotating field generation; 2: liquid to be measured; 3: magnetic bead; 4: inverted microscope objective. (**b**) Observed bead rotation frequency *vs*. field driving frequency. Below 9 Hz the bead rotation frequency matches that of the field; above 9 Hz, the bead rotates asynchronously, with frequency decreasing as the driving frequency increases. (**c**) Viscosity measurement of glycerol/water mixture solutions. The graph compares AMBR results in a magnetic field with 100 Hz driving frequency to published values and conventional (Ubbelohde) viscometer measurements of the same liquid. (**d**) AMBR microviscometer linear response to viscosity in prepared solutions of glycerol/water at 100 Hz driving frequency. Error bars represents standard deviation among three measurements.

**Figure A1:**
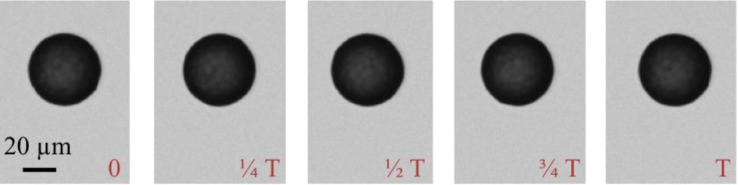
Images of the bead rotation at time 0, ^1^/_4_ T, ^1^/_4_ T, ^3^/_4_ T and T for 45 µm beads at 100 Hz driving frequency, where T is the bead rotation period. While the commercial beads look spherical and symmetrical by eye, the software can tell the subtle difference in shape and surface smoothness of the beads, so as to determine the bead rotation periods.

The glass slides were placed in a planar observation area confined within a controlled magnetic field. The latter was generated using orthogonal Helmholtz coils (Figure 1a). Viscosity measurements with AMBR microviscometer were conducted at 25 ± 1 °C. The magnetic field was measured with a 3-axis magnetic field probe (C-H3A-2m; Senis GmbH, Switzerland). The field strength was 2.7 mT and a driving frequency was as specified for each experiment, both of which were controlled with a custom LabVIEW program. Image stacks of bead rotations were recorded (Figure A1) during the experiment at a rate of 10 frames per second. Rotation periods of ten randomly selected beads were recorded for each sample, to accommodate the wide variance in commercial bead properties. The image stacks were analyzed using ImageJ, and a plot of image intensity *versus* image number was generated by ImageJ. The plot was imported into MATLAB, and the periodicity of the bead rotation was determined by applying a fast Fourier transform.

The viscosities of glycerol and water solutions at 25 °C were verified using an Ubbelohde viscometer. Briefly, 15 mL glycerol and water solution were poured into an Ubbelohde viscometer that was immersed in a water bath. The time that it took to pass through two calibrated marks on the viscometer was measured and used to determine the solution viscosity.

### 2.3. Preparation of Digestion Reaction Samples

In the digestion reactions, the restriction enzymes, the corresponding buffers, lambda DNA and nuclease free water were mixed and incubated at 37 °C for 1 h. After the reaction, the solutions were placed in a 25 °C water bath before being measured by the AMBR microviscometer.

### 2.4. Preparation of PCR Samples

All the reagents were added and mixed, and then distributed, 50 µL of the mixture to each tube. The tubes were capped during the reaction to prevent evaporation. Two tubes were used as the product of cycle 0, and the rest were put into a thermal cycler (Bio-Rad). The thermal cycling involved an initial denaturation at 95 °C for 30 s, followed by six amplification cycles. The thermal cycles were: 95 °C for 30 s (denaturation), 60 °C for 1 min (annealing), 72 °C for 5 min (extension). Then, the reactions were stopped and held at 4 °C. Two tubes of samples were taken out from the thermal cycler, and labeled as cycle 6. The rest of the samples underwent resumption of the reaction with an additional five cycles. This was repeated until a total of 41 cycles was completed for the last two tubes of samples. All the samples extracted from different cycles of the reaction were stored in a −20 °C freezer, and placed in a 25 °C water bath before AMBR measurement or gel electrophoresis.

### 2.5. Gel Electrophoresis

Gel electrophoresis was used to verify the DNA solution results measured by the AMBR microviscometer. A 0.8% agarose gel was prepared, and 1 µL reaction solution was diluted and loaded onto the gel. The gel electrophoresis was conducted in a 1× TBE buffer at 10 V/cm for 2 h. The fluorescent signal intensities of the 4500 bp bands were estimated with ImageJ.

## 3. Results and Discussion

### 3.1. Calibration of AMBR Viscometer

**Figure A2:**
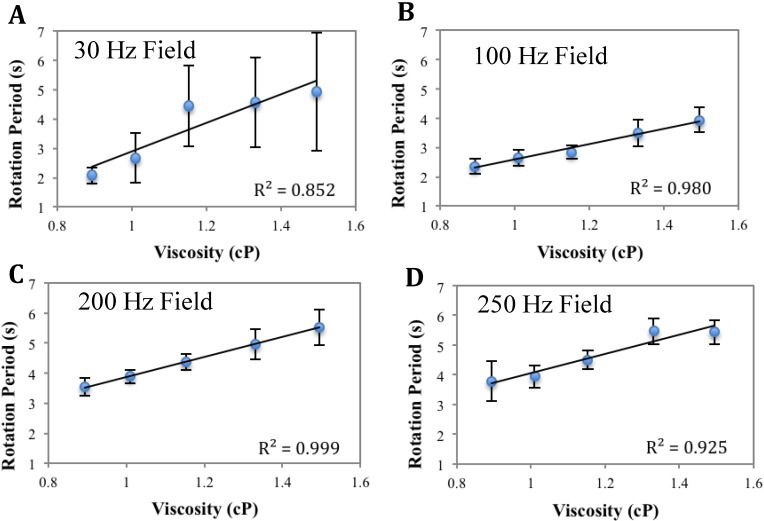
Calibration curves of 45µm bead at different driving frequencies. **A**: 30 Hz; **B**: 100 Hz; **C**: 200 Hz; **D**: 250 Hz. The critical frequency is at 10–15 Hz. The error bars represent the standard deviation among ten different beads in one measurement. The calibration curves yield good linearity consistently at frequencies away from the critical frequency, *i.e*., from 100 Hz to 250 Hz.

**Figure A3:**
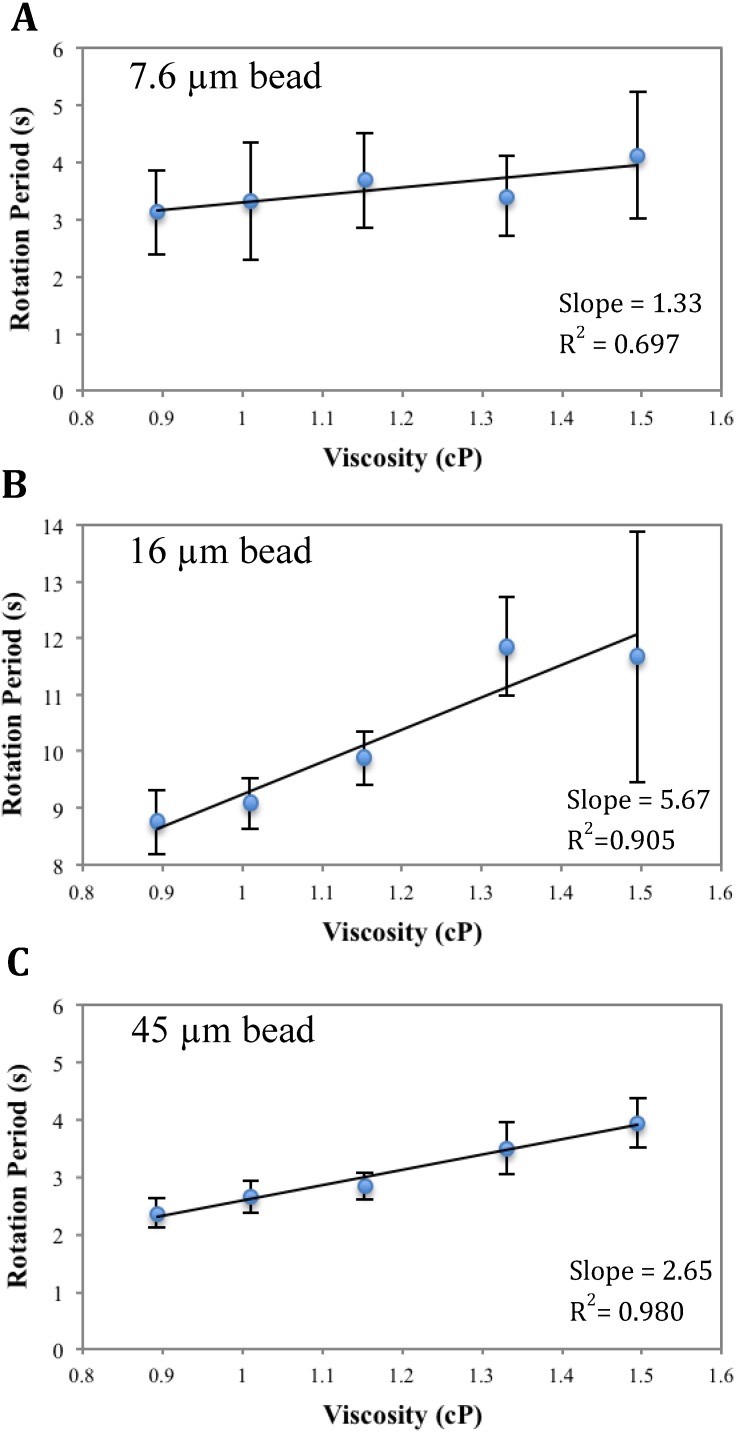
Calibration curves at 100 Hz using beads of different sizes. **A**: 7.6 µm; **B**: 16 µm; **C**: 45 µm. Error bars represents standard deviation among 10 beads in one measurement.

A linear relationship was found between the solution viscosity and the rotation period of the bead in the solution. A series of glycerol/water solutions with varying glycerol mass fraction were analyzed by the AMBR microviscometer and, in parallel, with an Ubbelohde viscometer [16]. The microviscometer results matched both the Ubbelohde viscosity values and the theoretically predicted values for the mixtures over a viscosity range from 0.89 to 2.8 cP (Figure 1c) [17]. A correlation curve relating the bead rotation period with the solution viscosity was constructed and yielded excellent uniformity (Figure 1d). The experimentally observed linear relationship between rotation period and viscosity agrees well with the theory developed for the paramagnetic AMBR system [18]. Additionally, the linear correlation is robust to variation in the magnetic field driving frequency (Figure A2), and for three different bead sizes tested, with the 45 µm beads giving optimal linear correlation results (Figure A3). However, we note that the linearity does not hold as well for a frequency close to the instability threshold as shown in Figure A2. Furthermore, the measurement of rotation period is not as reliable, because the jerky motion affects the image analysis. Therefore, we present our results at various driving frequencies in the Supplementary Materials, but chose to use only the higher driving frequency regime for the DNA measurements.

The observed linear correlation between solution viscosity and bead rotation period can be explained by the nonlinear magnetic oscillation theoretical framework [18,19,20,21,22]. At a low driving frequency, the bead rotates at the same rate as the driving magnetic field. However, as the driving frequency increases, the bead cannot overcome the viscous drag exerted by the surrounding fluid, and thus cannot follow the rotating magnetic field. The bead then rotates slower, and asynchronously, with respect to the driving magnetic field (Figure 1b) [18,20,22,23,24,25]. The nonlinear oscillation only occurs in the asynchronous regime. In a low Reynolds number environment, the force balance between the magnetic torque and the viscous drag yields the relationship between the bead rotation period and the solution viscosity. The effects of interaction between the magnetic bead and the solid surface can be neglected under the experimental conditions described in the Experimental Section. For a paramagnetic bead, the magnetic torque due to the induced magnetic dipole can be expressed as [18],

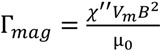
(1)
where *χ''* is the imaginary part of the magnetic susceptibility (which is frequency dependent), *V*_m_ is the volume of the bead’s magnetic content (*i.e*., the magnetic nanoparticles embedded in the bead), *B* is the strength of the driving magnetic field, and µ_0_ is the permeability of free space. The torque due to the viscous drag can be expressed as,

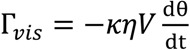
(2)
where θ is the arc length of the rotation, *κ* is the shape factor of the bead (*κ* = 6 for a sphere), *η* is the solution viscosity, and *V* is the volume of the magnetic bead. By combining Equations (1) and (2), the equation becomes, 
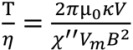
(3)
Therefore, in the asynchronous regime, the rotation period of a paramagnetic bead, under the rotating field of a given strength and frequency, is expected to be linearly proportional to the solution viscosity, *i.e*., T ∝ *η*. Our experimentally observed results confirm this theoretical relationship.

**Figure 2:**
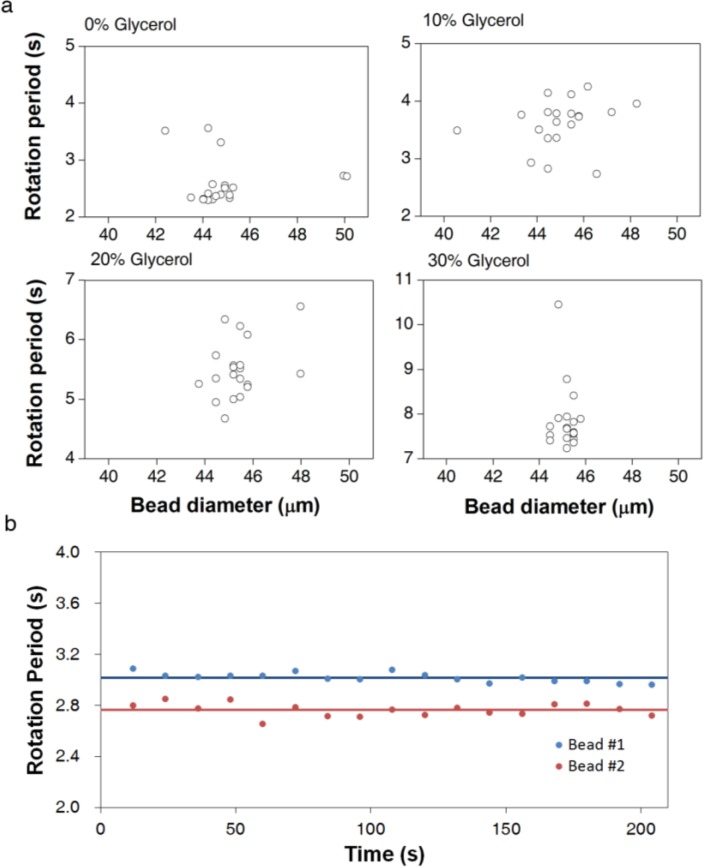
Reproducibility of AMBR viscosity measurements at 100 Hz driving frequency. (**a**) Rotation period measurement of 20 independent beads in the same solution plotted against the optically measured bead size of each bead. (**b**) The rotation periods of two examples of 45 µm beads observed over time in the same solution. The rotation periods are calculated over a 12 s period and plotted in the graph. The average values are for 17 sequential observations

To advance the practical utility of the asynchronous rotation method, we investigated the influence of the variation in bead properties on bead rotation periods. A relative standard deviation of approximately 10% is observed due to the variation in bead properties, such as size and magnetic content. As shown in Figure 2a, the rotation periods of 20 beads in the same solution do not show a clear bead-size dependency. Thus, bead-size non-uniformity is not the primary contributor to the variation in the rotation period measurement, despite the expected correlation in Equation (3). More likely, the bead magnetic properties, such as magnetic volume and susceptibility, are more significant for the inter-bead variation than is the size variation. The scattered pattern in Figure 2a supports the averaging over multiple beads in the construction of correlation curves and viscosity measurement experiments.

To confirm that inter-bead variation in the rotation period is primarily due to inherent bead properties, we measured the rotation period of the same bead continuously over time. The differences in rotation period over time are much smaller than the differences between two beads in the same experiment (Figure 2b). The relative standard deviation for a single bead over time is approximately 1%, 10 times smaller than the standard deviation in the rotation period among 10 beads. Therefore, the observed measurement error is smaller than the error caused by the bead non-uniformity. A wide variation in commercial bead properties has been observed before [26,27]; consequently, improved uniformity of bead magnetic character and size is expected to give better sensitivity in viscosity measurement.

### 3.2. Viscosity Measurement of DNA Aqueous Solutions

There is a linear relationship between the viscosity of common diagnostic reaction solutions and the concentration of DNA in those solutions. At a fixed temperature, the relationship between the solution viscosity, η, and the DNA concentration, C, for a very dilute solution can be expressed as η = η_0_(1 + C[η]), where η_0_ is the viscosity of the solvent and [η] is the intrinsic viscosity of the DNA product. This equation gives a linear correlation between the viscosity and the macromolecule concentration. The intrinsic viscosity increases with the molecular weight of dsDNA, and this correlation has been documented [15],

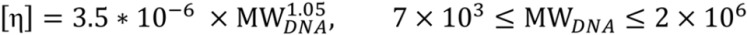


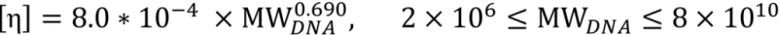

The linear relationship between the viscosity and the DNA concentration breaks down at very high molecular weight or high concentration due to the non-Newtonian property of the DNA solution [28].

**Figure 3:**
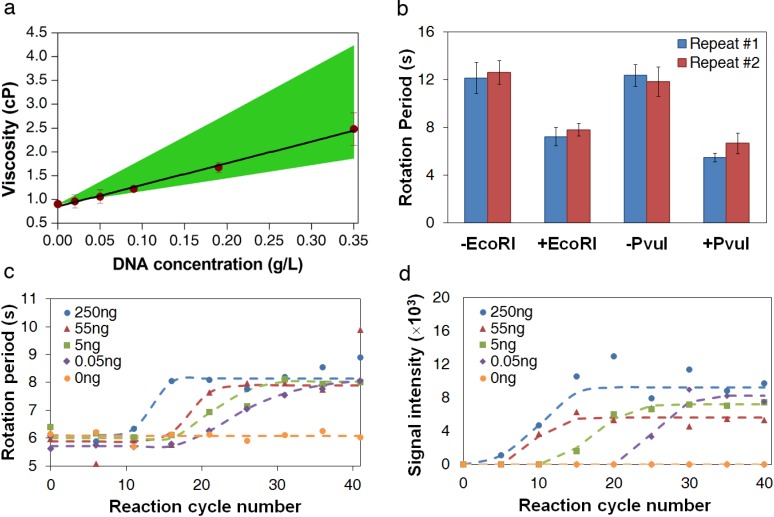
DNA measurement using AMBR microviscometer. (**a**) Viscosities of lambda DNA *Eco*RI digest solutions at different concentrations, as measured by AMBR microviscometer. The green area indicates the expected range of the viscosity calculated theoretically, assuming that only the longest (top range) or only the shortest (bottom range) DNA fragment size is present. Error bars represent standard deviation among 10 beads in one measurement. (**b**) Measurement of bead rotation period of pre- and post-digestion samples of lambda DNA by AMBR microviscometer. The field driving frequency is 150 Hz. The error bars show the standard deviation among 10 beads in each measurement. (**c**) Measurement of viscosity by bead rotation period in PCR reactions sampled every 5 cycles, starting from the 6th cycle. PCR reactions with initial DNA amounts of 0 ng, 0.05 ng, 5 ng, 55 ng, and 250 ng are shown. The reaction volumes are 50 µL each. The field driving frequency is 150 Hz, and the PCR product size is 4500 bp. Each point represents the mean value, observing ten beads. (**d**) Fluorescent signal intensities of the PCR product (4500 bp band) observed on a electrophoresis gel for the same samples measured in (**c**).

Digestion of lambda DNA with *Eco*RI has a variety of uses and performs a selective cleaving of DNA at a specific site, forming DNA fragments of length 3530, 4878, 5643, 5804, 7421 and 21,226 bp from DNA of original length of 48,502 bp. With the experimental relationship given in Figure 1d, we estimated the viscosities of the DNA *Eco*RI digest solutions, at different concentrations, using the measured bead rotation periods. A linear relationship was found between the solution viscosity and the DNA concentration (Table 1 and Figure 3a), confirming the assumption that these solutions were in the dilute solution regime. The viscosities of the DNA solutions measured using the AMBR microviscometer are within the theoretically estimated upper and lower bounds.

**Table 1:** Rotation periods and viscosities of lambda DNA *Eco*RI digest solutions at different DNA concentrations measured by AMBR microviscometer. The expected ranges of viscosities are calculated, assuming only the longest or shortest piece of DNA is present.

	Experimental Results		Expected Range	
DNA Conc. (g/L)	Rotation Period (s)	Viscosity (cP)	Min Viscosity (cP)	Max Viscosity (cP)
0.00	2.40 ± 0.24	0.90 ± 0.05	0.89	0.89
0.02	2.70 ± 0.64	0.96 ± 0.14	0.94	1.07
0.05	3.12 ± 0.62	1.06 ± 0.14	1.02	1.34
0.09	3.87 ± 0.21	1.22 ± 0.05	1.15	1.78
0.19	5.86 ± 0.49	1.67 ± 0.11	1.41	2.67
0.35	9.52 ± 1.53	2.48 ± 0.34	1.85	4.18

### 3.3. Measurement of DNA Reaction Progression

Measurements of restriction digestion samples confirm that the AMBR microviscometer is sensitive to viscosity changes caused by the DNA size changes. As shown in Figure 3b, a clear difference in bead rotation period can be seen between the digested and undigested lambda DNA solutions. Thus, the AMBR microviscometer can detect DNA sequence variation using a site-specific restriction endonuclease to essentially alter the solution viscosity.

**Figure A4:**
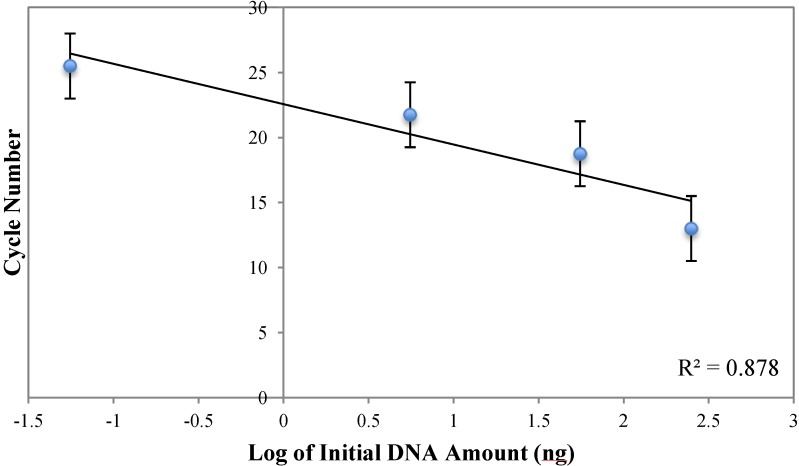
Plot of reaction cycle number *versus* log of initial DNA amount for the qPCR measurement by AMBR method. Error bars represent the uncertainty due to the AMBR measurement of every five cycles.

Measurements of PCR reaction samples over the course of the reaction show that the AMBR microviscometer can detect the formation of PCR products in real time. As expected, the reactions with the higher initial template concentration reach the maximum product concentration sooner than those with lower template concentrations (Figure 3c), and the plot of reaction cycle number *versus* log of initial DNA concentration yields a linear correlation (Figure A4). Comparing the AMBR measurements with the gel electrophoresis results on the same samples (Figure 3d) confirms that the viscosity-based method is approximately 5 cycles delayed, relative to gel electrophoresis detection.

Using commercial paramagnetic beads, the AMBR microviscometer is found to be sensitive to the viscosity changes associated with DNA reactions. The results on PCR, with a product size of 4500 bp, yield a 10% relative error in the rotation period measurement. The AMBR microviscometer should be able to detect PCR product sizes as low as 1000 bp, assuming a conversion of >95% of dNTPs to its polymerized product (*i.e*., 0.42 g/L final product concentration). However, this sensitivity can be further improved so as to meet the need of monitoring DNA reactions with smaller viscosity changes (e.g., PCRs with shorter DNA products) by optimizing the bead size, shape, and magnetic properties. Based on the 1% relative error observed for single bead measurements, over time, we predict that the AMBR microviscometer may be able to detect PCR with product size as low as 50 bp. By measuring the changes in viscosity of DNA solutions, our technique can measure the difference in molecular length for a known concentration or the difference in concentration for a known length.

## 4. Conclusions

In summary, the viscosity-based approach using an AMBR microviscometer introduces a new option for label-free DNA detection and for reaction monitoring. In the viscosity range of common DNA reactions, the measurement is completed within one minute, and a typical AMBR microviscometer set-up allows continuous, real-time measurement during the course of any reaction. This viscometer requires only a small amount of sample, and volumes in the picoliter range may be accessible if integrated into a microfluidic device. A laser-photodiode apparatus can easily replace the microscope detection setup used in this work, so as to make the measurement more cost-effective [29]. Although demonstrated with DNA solutions, the viscosity-based technology described here can be applied to any polymer reaction or degradation system. An improved understanding of the AMBR microviscometer performance in complex fluids may enable new applications, such as mapping the viscosity in living cells, understanding drug delivery mechanisms, and diagnosing blood clotting.

## Analysis of Figure A3

The slope of the curve as defined by Equation (3) increases with decreasing magnetic responsiveness of the magnetic beads and increasing bead sizes (Figure A3). The magnetic responsiveness takes into account a combination of factors, such as the volume of the magnetic content and the magnetic susceptibility, as defined in Equation (3). As expected, a smaller magnetic responsiveness gives a larger rotation period, under the same field, resulting in a larger slope (compare Figure A3(B) with the other two); also a bigger bead size improves the sensitivity of the viscometer, when the magnetic responsiveness is about the same (compare Figure A3(A, C)). These experimental observations are in agreement with the theoretical predictions indicated by Equation (3).
